# Continuous Flow UV-C Irradiation Effects on the Physicochemical Properties of *Aloe vera* Gel and Pitaya (S*tenocereus* spp.) Blend

**DOI:** 10.3390/foods9081068

**Published:** 2020-08-06

**Authors:** Carmen O. Meléndez-Pizarro, Arlet Calva-Quintana, José C. Espinoza-Hicks, Miguel Á. Sánchez-Madrigal, Armando Quintero-Ramos

**Affiliations:** Departamento de Investigación y Posgrado, Facultad de Ciencias Químicas, Universidad Autónoma de Chihuahua (UACH), Nuevo Campus Universitario, Circuito Universitario, Chihuahua CP 31125, Mexico; cmelende@uach.mx (C.O.M.-P.); a.calva95@gmail.com (A.C.-Q.); jhicks@uach.mx (J.C.E.-H.); msanchezm@uach.mx (M.Á.S.-M.)

**Keywords:** UV-C irradiation, *Aloe vera*, pitaya, nuclear magnetic resonance, betalains, polyphenols, blend

## Abstract

Physicochemical properties of a blend of 10% *Aloe vera* gel with 5% pitaya juice subjected to UV-C doses of 16.5, 27.7, and 40 mJ/cm^2^ were evaluated at pH 3.5 and 5.5. Unprocessed treatments were used as the control. The a* color parameter decreased and luminosity increased at pH 3.5. The decrease in the reddish color was consistent with the decrease in total betalains content and stabilized at pH 5.5. The NMR analyses of UV-C treatments showed changes in betalains signal patterns. Polyphenolics content was significantly reduced in the UV-C treatments at pH 5.5. UV-C processing decreased the antioxidant activity 1.25 times compared to unprocessed treatments. Total sugar content was reduced as the UV-C dose increased. Doses above 16.5 mJ/cm^2^ resulted in a higher simple sugar content at a pH 3.5. The UV-C continuous flow technology can be applied to stabilize betalains in *Aloe vera*–pitaya blends at a UV-C dose of 16.5 mJ/cm^2^ and pH 5.5.

## 1. Introduction

The market and the development of new functional beverages is growing rapidly. Different types of functional beverages are preferably consumed because they contain various bioactive compounds that provide health benefits. Generally, these compounds originate from fruits, vegetables, and plants [1]. Lately, *Aloe vera* (*A. vera*) plants have been used to develop trendy beverages because of their health benefits [2], which are attributed to the biological activity of various compounds found in the *A. vera* gel. Approximately 98.5–99.5% of the *A. vera* gel consists of water. The remaining solids are associated with different biologically active compounds. Among them are polysaccharides and their acetylated compounds, glycoproteins, phenolic compounds (anthraquinones, flavonoids, and flavonols), as well as enzymes, minerals, amino acids, sterols, saponins, and vitamins [3]. Despite the benefits of *A. vera* gel, the lack of flavor, color, and odor is a drawback that is frequently compensated by mixing the gel with fruits to enhance their sensorial characteristics. An exotic and colorful fruit with functional properties is the pitaya, a fruit obtained from the cacti *Stenocereus*. Pitaya fruit contains approximately 70% pulp and small seeds. Pitaya red juice has a high antioxidant potential since it contains natural pigments such as betalains, beta-carotene, and lycopene as well as vitamin E, minerals, phenolic compounds, and essential fatty acids [4]. Besides its attractive red color provided by betalains, the pitaya juice has a delicate and enjoyable taste. Therefore, a beverage containing *Aloe vera* gel and pitaya juice could result in an attractive product with satisfactory physicochemical, functional, and sensorial characteristics. However, the bioactivity of functional compounds in the resulting *Aloe vera*–pitaya blend could be negatively affected during the application of thermal methods [5] that are traditionally used to extend the shelf life of products. Among the *A. vera* gel compounds degraded by thermal processing are polysaccharides such as acemannan [6,7,8], while betalains [4,9] and polyphenols [10,11] are contained in both *A. vera* and pitaya. Therefore, moderate thermal treatment or non-thermal technologies are options to safely process these types of products with minimum chemical changes. The use of short-wave ultraviolet radiation (UV-C) alone or in combination with a moderate thermal treatment is an attractive technological alternative for food preservation [12]. Among the advantages of UV-C compared to other non-thermal methods is the absence of toxic residues, low energy consumption, and low operational costs [13,14]. The efficacy of UV-C in juices or blends is compromised by radiation penetration; therefore, the UV-C dose, the pH of the fluid, and optical properties of the liquid (absorption coefficient, turbidity, color) should be considered for successful treatment [15,16]. The use of UV-C radiation under appropriate conditions could represent an alternative to process a blend of *A. vera* gel with pitaya juice. However, data regarding this approach are scarce and the impact of such treatment on the physicochemical characteristics of an *A. vera* gel–pitaya juice blend is not well understood. The objective of this study was to evaluate the effect of UV-C radiation on the physicochemical characteristics of *A. vera*–pitaya blends at different pH values.

## 2. Materials and Methods 

### 2.1. Plant Materials and Reagents

Red pitaya (*Stenocereus* spp.) was obtained from the distribution center of Puebla, Mexico and *Aloe vera* plants were acquired from the greenhouse of the Chemical Sciences Department, University of Chihuahua. Ripe red pitayas were sorted and selected free from microbiological spoilage and physical damage. Homogeneous leaves of a four-year-old *A. vera* were used. A 200 ppm sodium hypochlorite solution was used to sanitize the pitayas and *A. vera* leaves. The pitayas were peeled and juiced; the juice was obtained using a Taurus^®^ Select Centurion 500 w domestic food processor. The juice was filtered using cotton cloths and stored at −18 °C. Meanwhile, the *A. vera* leaves were processed and the acibar was vertically drained for 1 h [17]. The gel was then separated from the *A. vera* skin leaves and homogenized in a blender (Phillips Electric Blender, Mexico), filtered, and stored at 4 °C for 1 h or less prior to the preparation of the blends. Gallic acid, 6-hydroxy-2-5-7-8-tetramethylchromo-2-carboxylic acid (Trolox), sodium bicarbonate (NaHCO_3_), Folin-Ciocalteu, glucose, fructose, saccharose, 2,2 diphenyl-1-picrylhydrazyl (DPPH), and sulfuric acid (H_2_SO_4_) were purchased from Sigma-Aldrich Co. (St. Louis, MO, USA). Analytical grade sodium chloride (NaCl) and sodium hydroxide (NaOH) were procured from Fermont (Monterrey, Mexico). HPLC-grade acetonitrile and water were secured from J.T. Baker (Mexico).

### 2.2. Aloe vera–Pitaya Blend

To prepare the *Aloe vera*–pitaya blend, the *A. vera* gel obtained was diluted at 10% with water and 5% of the pitaya juice was added. Sucrose (5%) was used as a sweetener. Two *A. vera*–pitaya blend batches at two different pH values (3.5 and 5.5) were obtained, adding acid or basic components to reach the desired pH value. The *A. vera*–pitaya blends were stored at 4 °C for 2 h or less prior to the UV-C irradiation.

### 2.3. UV-C Light Processing

Each batch was subjected to different UV-C irradiation doses which was varied according to the flow rate (37.85, 56.78, and 94.63 L per hour), resulting in residence times of 16.57 ± 0.02, 11.05 ± 0.14, and 6.63 ± 0.12 s, respectively, in a continuous-flow CiderSure 3500 commercial UV unit (FPE Inc., Macedon, NY, USA). All treatments were performed in duplicate at room temperature (20 °C). The unit was sanitized with 200 ppm of hypochlorite solution and rinsed with water before and after each treatment. A thin film of *A. vera*–pitaya blend was irradiated at 254 nm with eight low-pressure mercury lamps. The irradiance was controlled every 50 ms by two UVX-25 sensors (UVP, Inc., Upland, CA, USA). The real irradiance was obtained by considering the mean of the irradiance obtained for each sensor. Exposure times were obtained from the flow rate for each treatment. The UV-C dose was calculated using Equation (1) [18]:

(1) $$UV\;dose\left(\frac{mJ}{cm^{2}}\right)=Irradiance\times Exposure\;time.$$

Unprocessed (UP) and UV-C processed samples were analyzed immediately after irradiation to measure their physicochemical features.

### 2.4. Physicochemical and Optical Properties of the Aloe vera-Pitaya Blend

The *A. vera*–pitaya blend before pH adjustment was characterized for optical and physicochemical properties. The optical properties of the blend were obtained using a UV-Vis spectrophotometer (Lambda 25, Perkin Elmer, Waltham, MA, USA) [19]. The absorption coefficient (α) was obtained at 254 nm and calculated from the Lambert–Beer law equation. Demountable fused quartz cuvettes (FireflySci, Inc., NY, USA) with path lengths of 0.1, 0.2, 0.5, and 1.0 mm were used. The absorption coefficient, log base 10, is expressed in cm−^1^ and was determined using the slope of the line equation and path lengths of the cuvettes. Using the reciprocal of the absorption coefficient (1/α), the penetration depth (λ) was obtained in cm. Turbidity was measured using a micro-turbidimeter 100 instrument (Micro 100 Hf, Scientific, Inc., Fort Myers, FL, USA). Results were expressed as Nephelometric Turbidity Units (NTU). Soluble solids were measured using a refractometer (Abbe, American Optical Corporation, NY, USA). All measurements were performed in triplicate and the results are reported as mean ± standard deviation (SD).

### 2.5. Analytical Methods

Total polyphenol content (TPC), total antioxidant activity (TAA), and total sugar content (TS) were quantified in UP and UV-C-irradiated *A. vera*–pitaya blend samples by spectrophotometric methods. A UV-Vis spectrophotometer was used (Perkin Elmer Lambda 25, Waltham, MA, USA). TPC was determined with the Folin–Ciocalteu method [20], with some modifications. The blend (30 µL), deionized water (3 mL), and Folin–Ciocalteu (200 µL) were mixed for 10 min at room temperature. A 20% Na_2_CO_3_ solution was added (600 µL) and this mixture was shaken for 10 s, incubated in a water bath at 40 °C for 20 min, and then cooled. Subsequently, the samples were read at an absorbance of 760 nm in triplicate using gallic acid as the standard. The results were expressed as milligrams of gallic acid equivalents per gram of blend dry mass (mg GAE/100 g dm). The total antioxidant activity was determined according to the free radical method using 2,2 diphenyl-1-picrylhydrazyl (DPPH˙) [21]. The blend (100 µL) and 3.9 mL of the 100 µM DPPH˙ solution were mixed and allowed to stand in the dark for 3 h. The absorbance was then measured at 517 nm. The calibration curve was obtained using Trolox as the standard and methanol as the blank. Measurements were made in triplicate and the results were expressed as µmol Trolox equivalents per gram of blend dry mass (µmol TE/g dm). Total sugars were quantified according to the phenol-sulphuric method [22]. Glucose was used as the standard for the calibration curve. The determinations were performed in triplicate and the glucose content was expressed as mg/g dry mass (dm) of the *A. vera*–pitaya blend.

### 2.6. Aloin A Content

The aloin A content of UP and UV-C irradiated samples was determined by high resolution liquid chromatography (UHPLC) according to Bozzi et al. [23], with some modifications. A Thermo Scientific Dionex Ultimate 300 (Sunnyvale, CA, USA) equipped with a quaternary pump, autosampler, and diode array detector (DAD) model ProStar 410 (Varian, Palo Alto, CA) was used. Samples were filtered through 0.45 µm nylon filters. The separation was performed by adding 0.1% v/v acetic acid to the mobile phase. The injection volume was 25 µL, the flow rate was 1.0 mL/min at 50 °C and a column AcclaimTM 120-C18 (120 Å, 4.6 × 150 mm) and a wavelength of 297 nm were used. High-purity aloin A (Sigma-Aldrich, St. Louis, MO, USA) was applied as the standard. Aloin A contents were presented as the mean of triplicate analyses with standard deviation and expressed as mg/L of blend.

### 2.7. Betalain Quantification

The betalains content in the *A. vera*–pitaya blends was assessed according to Castellanos–Santiago and Yahia [24] and a UV-Vis spectrophotometer (Perkin Elmer) was employed. The betacyanin content (BC) and betaxanthin content (BX) were calculated using the following Equation (2):

(2) $$B\;\left[\frac{mg}{g}\right]=\left[A*DF*MW*\frac{VD}{\varepsilon *L*Wd}\right],$$

where A corresponds to the absorbance of betacyanin and betaxanthin at maximum wavelengths of 535 and 483 nm, respectively. DF is the dilution factor and MW is the molecular weight (g/mol), which was 550 g/mol for betacyanin and 308 g/mol for betaxanthin. VD is the volume of the solution (mL) and ε is the corresponding molar absorptivity coefficient in water, for betanin (60,000 mol/L cm) and betaxanthin (48,000 mol/L cm). L is the cell path length (1 cm) and Wd corresponds to the dry weight (g) of the product. Measurements were carried out in triplicate. The results were expressed as mg BC/100 g dm and mg BX/100 g dm. The total betalain content (BT) was calculated as the sum of betacyanin and betaxanthin contents.

### 2.8. Betacyanin Retention

For the retention of betacyanin and betaxanthin in the *A. vera*–pitaya blend, a method described by Cai et al. [25] was carried out. Filtered samples (0.45 µm nylon filters) were injected (20 µL) into a UHPLC Dionex Ultimate 3000 (Thermo Scientific, Sunnyvale, CA, USA) equipped with an autosampler, a diode array detector model ProStar 410 (Varian, Palo Alto, CA), and a Thermo Scientific C18-reversed phase column with a particle size of 0.5 µm. Mobile phase A was methanol/0.05 M KH_2_PO_4_ (18:82 v/v) at a pH of 2.75 with phosphoric acid. Mobile phase B consisted of methanol; the elution gradient was from 100% to 80% of A and 20% of B in 20 min at a flow rate of 1 mL/min. The detection was carried out at 481 and 536 nm for betaxanthin and betacyanin, respectively. The retention (%) was calculated using Equation (3):

(3) $$\%\;Retention=\frac{A}{A_{0}}*\;100,$$

where A is the area of the UV-C irradiated sample and A_0_ corresponds to the unprocessed (UP) sample area. The results were expressed as % retention for both betacyanin and betaxanthin. Measurements were carried out in duplicate and mean values and standard deviations reported.

### 2.9. Simple Sugar Content

Simple sugar content was determined by high-performance anion-exchange chromatography with pulsed amperometric detection (HPAE-PAD), a modified method of Bozzi et al. [23]. The precipitated polysaccharides (2 mg) were dissolved in distilled water (2 mL) and stirred for 24 h at 4 °C. The solution was filtered (0.45 µm) and analyzed by a Thermo Scientific Dionex ICS-5000+ system (Sunnyvale, CA, USA) equipped with an autosampler and an electrochemical detector; a gold working electrode and a silver chloride reference electrode were used. Sugar separation was achieved on an anion-exchange resin CarboPac PA1 column with a pre-column (250 × 4 mm and 50 × 4 mm, respectively). As eluents, water and 150 mM NaOH were used as mobile phases A and B, respectively. The elution gradient employed was: 0–15 min with 100% B, 15–25 min linear gradient of 0–88% for A, and 30–50 min with 88% of A and 12% of B. Glucose, fructose, and sucrose were used as standards. All measurements were performed in triplicate and the content of simple sugars was expressed as mg/g dry mass of the blend (mean value ± SD).

### 2.10. Color Characteristics

Color parameters (L*, a*, b*) of unprocessed (UP) and *Aloe vera*–pitaya blends treated with UV-C irradiation were determined using a calibrated colorimeter (Minolta CR 400/410, Konica Inc., Japan) calibrated with a tile standard with values of X = 94.9, y = 0.3185, and x = 0.3124. The blend was placed in a Petri dish to measure L* (luminosity, white (100) – black (0)), a* (green (-) – red (+)), and b* (blue (-) – yellow (+)) color parameters on the Hunter scale. The measurements were performed four times in triplicate. The mean values with SD were reported.

### 2.11. NMR Analysis of Concentrated Betacyanins 

Unprocessed and UV-C processed treatments at 16.5 mJ/cm^2^UV-C dose adjusted at pH 3.5 (30 mL of each) were concentrated at 40 °C under reduced pressure to evaporate the water. The elimination of sugars was carried out using C18-reversed phase cartridges (Waters Sep-Pak C18 – 5 g). The sorbent material was activated with MeOH and then rinsed with acidified water (pH = 3, with trifluoroacetic acid (TFA)). The concentrated samples (UP and UV-Dose) were resuspended in 1 mL of water at pH 3 (adjusted with 0.1 N HCl) and then applied to the sorbent. The sugar present in the sample was eluted with acidified water (pH = 3 with TFA). Betacyanins were eluted adding acidified MeOH (95/5 MeOH/pH 2 water, v/v). The colored fraction was concentrated in vacuo at 40 °C until dryness [26]. The purified samples were resuspended in 0.6 mL of D_2_O with 0.1 mM of TMSP-d_4_ as an internal standard and 2 mM of NaN_3_ and then analyzed by NMR using a 400 MHz Bruker Advance III spectrometer. All the samples were acquired using the WATERSUP experiment with the *noesygppr1d* pulse sequence, the number of scans was 256, and the dummy scans 8. All measurements were performed at 25 °C and the calibration of pulse and O1P was realized before the analysis.

### 2.12. Experimental Design and Statistical Analysis

A completely randomized factorial design was used (3 × 2) to determine the influence of the UV-C dose (16.5, 27.7, and 40 mJ/cm^2^) and pH (3.5 and 5.5) on the *A. vera*–pitaya blend. As a control, an unprocessed (UP) treatment was used. The data obtained from the various experiments were subjected to a contrast analysis as well as an analysis of variance (ANOVA) and mean difference by Tukey’s test at *p* < 0.05 using Microsoft Excel software (Microsoft Excel software version 16 Minitab 15 program (Minitab, 2010, State Collage, PA, USA)) [27]. 

## 3. Results and Discussion

### 3.1. Chemical Characterization and Optical Properties of Aloe vera–Pitaya (Stenocereus spp.) Blend 

The blend had a pH of 4.48 ± 0.01 and 5 °Bx as soluble solids. The optical properties featured an absorption coefficient of 2.35 cm^−1^, a penetration depth of 0.43 cm, and turbidity of 33.78 ± 1.94 NTU. It was reported that an absorption coefficient of less than 15 cm⁻^1^ is desirable to ensure a reduction of 5 log of *E. coli* K12 for fruit juices [28]. The turbidity obtained was higher than that reported by Rodríguez–Rodríguez et al. [11] for UV-irradiated *A. vera* gel–water blends (21.50 NTU). The optical properties of the *A. vera*–pitaya blend suggests an effective UV-C irradiation treatment [29]. Concerning the color features, the L*, a*, and b* parameters were 28.68 ± 0.30, 16.24 ± 0.36, and 7.51 ± 0.14, respectively, showing a reddish tone derived from pitaya. The pigments betacyanin and betaxanthine were 47 ± 1.0 and 59 ± 1.0 mg/100 g dm, respectively. The total polyphenol content was 76.15 ± 4.6 mg EAG/100 g dm. The aloin A content, originating from *A. vera*, was 5.62 ± 1.13 mg/100 g dm. The total antioxidant activity content was 179.71 ± 6 µmol TE/g dm and total sugars and sucrose content was 823.31 ± 19.5 mg/g dm and 608.73 ± 40.43 mg/100 g dm, respectively. 

### 3.2. Color

**Figure 1:**
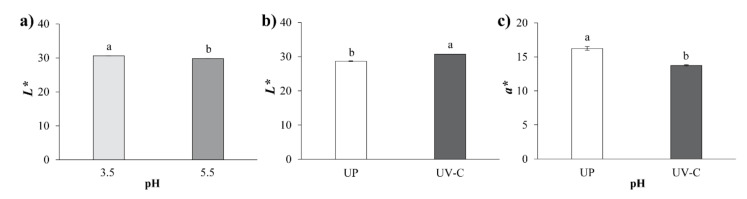
Effects on color parameters of *Aloe vera*–pitaya blends subjected to UV-C treatments and different pH values (**a**) Effect of pH on L* parameter. (**b**) Effect on the L* parameter of UV-C treatments compared to UP (unprocessed) treatments. (**c**) Effect on the a* parameter of UV-C treatments compared to UP (unprocessed) treatments. For each figure panel, the different letters indicate significant differences between the treatments at *p* < 0.05.

Color is a fundamental characteristic of juices and beverages and it is desirable that the preservation treatments do not affect it. Table 1 shows the contrast analysis for the color parameters of the *A. vera*–pitaya blend subjected to different pH and UV-C irradiation doses. It was observed that pH and UV-C treatments significantly affected L*, a*, and b* color parameters. However, the UV-C dose did not have any effect (*p* > 0.05). The lightness, concerning the L* parameter, was slightly lower (*p* < 0.05) at pH 5.5 than at pH 3.5 (Figure 1a) and an increase was also observed (*p* < 0.05) upon UV-C irradiation (Figure 1b). This could be attributed to a less reddish tone related to the changes in the betalain content, which increases L*. Betalain degradation, while increases in L* values during the processing of milk supplemented with betalains was reported by Güneser [9]. Regarding the a* parameter, relating to green (-) and red (+) tones, positive values were obtained, indicating redness, which was provided by the betalains contained in the pitaya. Table 2 shows a significant reduction in the a* parameter at pH 3.5. The higher a* value at pH 5.5 is in agreement with Stintzing et al. [30], who determined that the highest color stability of betalains from pitaya was in the pH range of 5–7. Betalain-type pigments from pitaya are stable at pH values between 4 and 6 [31]. The a* parameter decreased significantly upon UV-C treatment compared to that of the unprocessed treatment (Figure 1c). This decrease is associated with the effect of UV-C radiation on betalains; the color is provided by the double bonds and aromatic rings of the betalains, which absorb UV-C radiation [15,32]. With respect to the b* parameter, with a blue (−) and yellow (+) tendency, the positive values obtained in all treatments indicate yellowish tones. Table 1 shows the interactive effect between pH and treatments (UP and UV-C). Table 2 displays that only the UV-C treatment at pH 3.5 had a significant effect on the b* parameter of the blend, indicating a yellowish color tendency; therefore, lightness was slightly increased.

### 3.3. Effect of UV-C Procesing on Betalains, Betacyanins, and Betaxanthines

**Figure 2:**
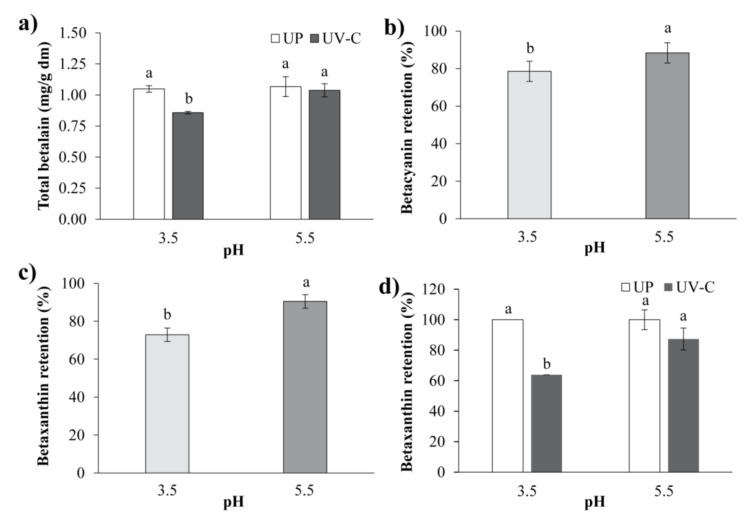
Effects on betalain-type pigments of the *Aloe vera*–pitaya blend. (**a**) Effect of pH on total betalains content of unprocessed (UP) and UV-C irradiated treatments. (**b**) Effect of pH on betacyanin retention (%BC). (**c**) Effect of pH on betaxanthin retention. (**d**) Effect of pH on betaxanthin retention (%BX) of unprocessed (UP) and UV-C irradiated treatments. For each figure panel, the different letters indicate significant differences between the treatments at *p* < 0.05.

Betalains are a group of reddish natural pigments that provide the characteristic color to pitaya (*Stenocereus* spp.). The betalains (BT) content of the blend was affected (*p* < 0.05) by pH and UV-C processing (Table 1). It was observed that pH did not produce changes in BT content for UP treatments. The highest BT values were obtained at a pH of 5.5 for UP and UV-C treatment. Although the UV-C irradiation dose did not significantly affect the BT content, Figure 2a shows that upon UV-C irradiation, the betalain content decreased by 10.45% compared to UP treatment at pH 3.5. Betalains of pitaya were stable in the pH range of 3.7 to 5.5 [33].

Betalains, which include betacyanins and betaxanthines, are correlated with red and yellow color. The retention of betacyanins (%BC) in the blend was affected (*p* < 0.05) by UV-C processing and pH (Table 1). Figure 2b shows the highest percent betacyanins retention at pH 5.5. Tang and Norziah [34] reported that betacyanins were more stable at pH values between 5 and 6. This agrees with Woo et al. [32] and Wong and Siow [35], who observed a better content of betacyanins from red-fleshed dragon fruit (*Hylocereus polyrhizus*) at pH 5. In addition, UV-C irradiation significantly decreased the %BC (22.06%) compared to UP treatment.

The stability of betacyanins in dragon fruit (*Hylocereus polyrhizus)* was affected by UV light [32,35], which reduces its red tone. Degradation of betacyanins produces colorless ciclo-dopa 5-O-β glucoside and bright yellow betalamic acid [36]. Regarding the impact of UV-C treatment on the percent retention of betaxanthines (%BX) in the blend, UV-C processing and pH and combinations thereof noticeably affected its individual form (Table 1). The UV-C treatments decreased (*p* < 0.05) the %BX content, specifically (*p* < 0.05) at pH 3.5 (Figure 2c,d). Cai et al. [37] reported that aqueous solutions containing betaxanthins were considerably stable at pH 5.5. The percent retention of betaxanthins was higher than that of betacyanins after UV-C treatment. Betaxanthins were more stable than betacyanins under certain treatments, such as storage at 60 °C and in vitro digestion [38].

### 3.4. NMR Analysis of UV-C Processed and Unprocessed Betalains Samples

**Figure 3:**
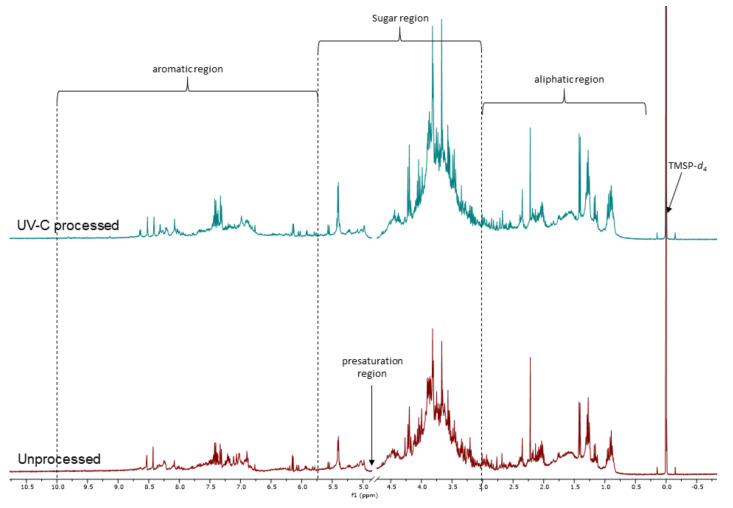
^1^H NMR (400 MHz, D_2_O) spectra of UV-C processed (dose 16.5 ± 0.21 mJ/cm^2^ and pH 3.5) and unprocessed (pH 3.5) samples.

**Figure 4:**
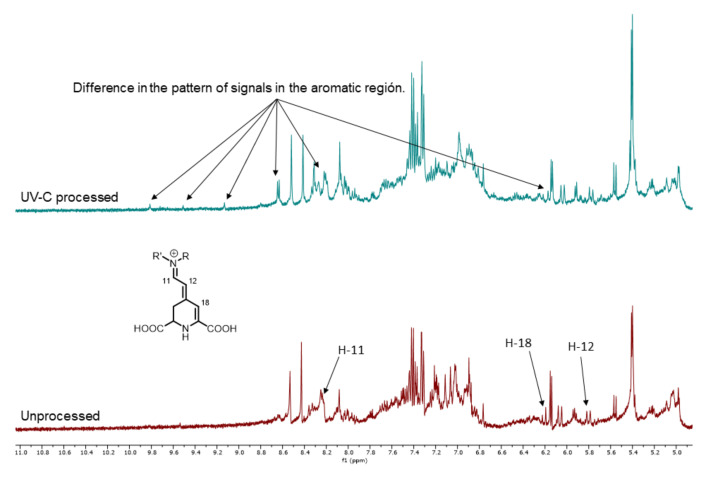
^1^H NMR (400 MHz, D_2_O) expansion on the aromatic region in spectra for UV-C processed (dose 16.5 ± 0.21 mJ/cm^2^ and pH 3.5) and unprocessed (pH 3.5) samples.

Figure 3 shows the NMR spectra of the isolated colored fractions obtained from the UV-C process (pH 3.5 and UV-C dose of 16.5 mJ/cm^2^) and unprocessed treatments (pH 3.5). It can be seen that the aromatic region (5.6–9.0 ppm) displays modifications in the signal profile. Figure 4 presents the expansion of the aromatic region. The spectrum of the sample without treatment shows characteristic signals of the base structure of pigments corresponding to betacyanins. The signals were compared to those reported by Stintzing et al. [30], where the chemical shifts of 6 isolated betalains from pitaya (*H. polyrhizus*) were observed. It was reported for the betalains that vinylic hydrogens H-11, H-12, and H-18 displayed signals at 8.10–8.19, 5.80–5.84, and 6.18–6.22 ppm, respectively. A similar signal pattern was found for the UP treatment. The NMR spectrum shows a different signal pattern for the UV-C process. This could be indicative of the degradation of this type of compound, mainly by oxidation [39]. This result agrees with the decrease in the retention of betacyanins obtained by UHPLC.

### 3.5. Total Polyphenols

**Figure 5:**
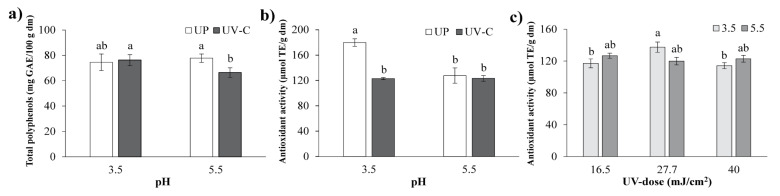
Total polyphenols and total antioxidant activity of the *Aloe vera*–pitaya blend. (**a**) Effect of pH on total polyphenol content in unprocessed (UP) and UV-C irradiated treatments. (**b**) Effect of pH on total antioxidant activity in unprocessed (UP) and UV-C irradiated treatments. (**c**) Effect of pH at different UV-C doses on total antioxidant activity. For each figure panel, the different letters indicate significant differences between the treatments at *p* < 0.05.

The total polyphenol (TP) content in the blend was affected (*p* < 0.05) by pH, but did not change significantly after UV-C treatment (Table 1). Figure 5a exhibits, compared to the UP treatments, a decrease in TP content for the UV-C treatments at pH 5.5. Polyphenols are affected by reaction conditions and type of processing. In addition, they are sensitive to pH changes mainly because of their phenolic and carboxylic groups. Better stability of these compounds is observed in acidic conditions as the finger millet polyphenol content decreases as the pH increases [40]. Although pH is one of the main factors that influences the stability of phenolic compounds, other factors such as the compound structure have an important effect on their stability [41]. Results showed that the phenolic content was not affected upon UV-C irradiation, which is in agreement with results reported for pomegranate juice [42], orange juice [43], apple juice [44], orange and carrot juice blends [45], and a turbid carrot–orange juice blend [46] subjected to UV-C irradiation. This behavior was attributed to the short exposure time to UV-C light, thereby avoiding the photo-oxidation of polyphenols.

### 3.6. Total Antioxidant Activity

The total antioxidant activity (TAA) of the blend was affected by pH and UV-C irradiation with respect to their linear and interaction effects (Table 1). The UV-C treatment decreased the TAA compared to the UP treatments without changing pH (Figure 5b).

The highest TAA was for UP treatments at pH 3.5. The UV-C dose and pH significantly affected the TAA in the blend (Table 1). The effect of different UV-C doses on TAA at the two adjusted pH values is presented in Figure 5c. A quadratic tendency was obtained at a pH of 3.5, achieving the highest TAA content *(p* < 0.05) at 27.7 ± 0.93 mJ/cm^2^, with a decrease at the highest UV-C dose (40 ± 0.48 mJ/cm^2^). At pH 5.5, the lowest TAA content was observed, without significant changes at any UV-C dose. This behavior was similar to that observed for the total polyphenol compounds of UV-C treatments. The total polyphenol content was correlated with TAA, influencing its properties [47]. The TAA reduction at a UV-C irradiation dose > 24.2 mJ/cm^2^ was reported for *A. vera* gel blends [11].

### 3.7. Total Sugars

The linear pH effect together with UV irradiation as well as the interaction of UP with UV-C treatments markedly affected the total sugar (TS) content (Table 1). The TS content did not change much without irradiation at both pH values (Table 3). The only significant difference observed was with UV-C irradiation. The UV-C treated blend at pH 3.5 had a lower TS content (*p* < 0.05) compared to that at pH 5.5. Regarding the effect of UV-C dose and pH, an increase in TS was observed as the UV-C dose increased. Also, the lowest TS values were determined at pH 3.5 and a dose ≤ 27.7 ± 0.93 mJ/cm^2^, while in blends at pH 5.5 and at doses > 27.7 ± 0.93 mJ/cm^2^, the TS content decreased slightly (*p* < 0.05). Rodríguez-Gonzalez et al. [7] and Rodríguez-Rodríguez et al. [8] attributed changes in TS content in *A. vera* gel to the pH and the effect of processing combination. 

### 3.8. Free Sugar Content

Glucose is the main monosaccharide present in pitaya [48] and *A. vera* [8]. Table 1 shows the effect of UV-C irradiation and pH on the free sugar content of the *Aloe vera*–pitaya blend. Glucose, fructose, and sucrose content were considerably affected by pH, UV-C irradiation treatment, and dose. Table 3 shows the glucose content of the blends for UP and UV-C treatments at two pH values.

It is observed that the glucose content was higher upon UV-C irradiation compared to UP. In addition, the highest glucose content (*p* < 0.05) was achieved at both pH for UV-C irradiated treatments. The glucose content was significantly higher in the UV-C irradiated treatments at pH 3.5. Furthermore, both the irradiation dose and pH interactively affected the glucose content (Table 1). At pH 3.5 and a UV-C dose > 27.7 ± 0.93 mJ/cm^2^, the glucose content increased, while a pH of 5.5 and a dose of 16.5 ± 0.21 mJ/cm^2^ resulted in the lowest glucose content (*p* < 0.05), increasing at higher UV-C doses of 27.7 mJ/cm^2^ (Table 3). Rodríguez-Rodríguez et al. [8] determined that UV-C doses > 24.2 mJ/cm^2^ increased the glucose content in the 10% *A. vera* gel, which was attributed to the irradiation-induced hydrolysis of oligo and polysaccharides (acemannan) contained in the *A. vera* gel. Moreover, Minjares-Fuentes and Femenia [49] related the glucose increase to acemannan degradation. Likewise, increases in glucose and fructose in apple juice at UV-C irradiation > 40 mJ/cm^2^ were reported by Islam et al. [50].

The content of fructose and glucose were noticeably affected by UV-C processing, doses, and pH (Table 1). The interaction of pH between UP and UV-C treatments of the blend showed an increase in fructose content for UV-C treatments (Table 3). Here, the fructose content decreased at pH 5.5. Regarding the interactive effect between UV C dose and pH, it was observed that UV-C irradiation doses > 16.5 ± 0.21 mJ/cm^2^ resulted in a high fructose content in the blend at pH 5.5, without showing any effects at pH 3.5 (Table 3). Rodríguez-Rodríguez et al. [8] reported a similar tendency for 10% *A. vera* gel. The sucrose content (SC) was higher (*p* < 0.05) in the UV-C irradiated treatments than in the UP treatments at both pH values (Table 3). The sucrose content increases as UV-C doses increase (16.5 mJ/cm^2^) and the highest sucrose content was observed at pH 3.5 (Table 3). The sucrose content in the blend originates from pitaya juice [51] and oligofructans or fructans inulin-type are contained in the *Aloe vera* gel [52,53]. Oligofructans hydrolysis at elevated UV-C irradiation doses generated increments in this disaccharide. Similar results were reported by Rodríguez-Rodríguez et al. [8].

### 3.9. Aloin A

The aloin A concentration in the *A. vera*–pitaya blends for the different treatments ranged from 2.58 ± 0.46 to 3.99 ± 0.12 mg/L (Table 3). These values are below the maximum permitted by the International Aloe Science Council. The aloin A content was not markedly affected by pH nor by UV-C irradiation in comparison to the unprocessed treatments (Table 1). Similar observations were reported by Rodríguez-Rodríguez et al. [11], who attributed the aloin A changes to the pH effect. Other authors reported that aloin A stability is pH-dependent, with high stability at low pH values (<3) and decreasing stability at pH > 5 [10]. Although this pH range is similar to that used in this research, the aloin A content was not affected.

## 4. Conclusions

The physicochemical changes of the *A. vera*–pitaya blend during the processing with continuous flow UV-C technology are dependent on the pH value of the blend. At pH 3.5, the total polyphenol content increased, while the total antioxidant activity and total betalains content decreased compared to the unprocessed *A. vera*–pitaya blend. On the other hand, at a pH value of 5.5, the UV-C irradiation treatments did not show changes in total betalains compared with unprocessed blends. The structural changes in betalains were corroborated by NMR. The betalains changes were associated with luminosity and the a* color parameter was indicative of the redness tone of the blend. Also, this pH caused increases in simple sugars content (glucose, fructose, and sucrose).

The reddish pigmentation of the *A vera*–pitaya blend is dependent on pH. The UV-C dose did not cause changes for most of the bioactive compounds of the blend.

## Figures and Tables

**Table 1:** Contrast analysis of treatment effects on the physicochemical properties of *Aloe vera*–pitaya blends at different pH values.

Source	DF	Sum of Square												
		TP	Aloin A	TAA	*L* *	*a* *	*b* *	Glucose	Fructose	Sucrose	TS	BT	BC%	BX%
Model	7	536.767	10.702	44422.98 *	16.318 *	30.857 *	7.698 *	45.847 *	30.744 *	360.263 *	6272.48 *	1492.320 *	2106.460 *	3678.577 *
pH	1	251.368 *	1.272	304.105 *	2.5149 *	7.617 *	3.875 *	8.2661 *	4.89459 *	65.771 *	876.38 *	772.490 *	385.926*	1238.512
UP vs UV-C	1	61.111	0.492	33038.14 *	12.434 *	18.884 *	2.816 *	25.703 *	13.2101 *	234.548 *	124.790	366.807 *	1459.048 *	1786.446 *
pH *UP vs UV-C	1	166.288 *	0.809	7675.694 *	0.719	3.279	0.906 *	0.8752 *	0.5102 *	23.614 *	2870.04 *	193.601 *	128.67	412.837 *
D	2	51.535	6.114	562.874 *	0.338	0.840	0.014	6.6157 *	9.2903 *	30.297 *	24.088	94.847	130.441	165.546
pH*D	2	6.464	2.016	2842.172 *	0.312	0.236	0.088	4.3878 *	2.8383 *	6.033 *	2377.17 *	64.573	2.402	75.236
Error	8	220.604	9.784	392.659	1.632	8.305	0.373	0.0201	0.2542	0.809	482.102	307.833	343.083	227.379

* Significance level at *p* < 0.05. UP, unprocessed treatment; UV-C, UV treatment; D; UV irradiation dose; TP, total polyphenols; TAA, total antioxidant activity; L*, lightness (−/+); a*, greenness/redness (−/+); b*, blueness/yellowness (−/+);TS, total sugars; BT, betalains; BC; betacyanin; BX, betaxanthin.

**Table 2:** Effects of the UV-C radiation and pH on color parameters of *Aloe vera–*pitaya blends.

Treatment/pH	L *	a *	b *
UP/3.5	28.71 ± 0.54 ^b^	16.34 ± 0.62 ^a^	7.59 ± 0.15 ^b^
UP/5.5	28.65 ± 0.18 ^b^	16.15 ± 0.14 ^ab^	7.43 ± 0.07 ^b^
D1-UV/3.5	31.24 ± 0.32 ^a^	12.97 ± 1.44 ^ab^	8.97 ± 0.33 ^a^
D1-UV/5.5	30.37 ± 0.08 ^ab^	14.56 ± 0.20 ^ab^	7.94 ± 0.01 ^b^
D2-UV/3.5	31.61 ± 0.86 ^a^	12.26 ± 1.91^b^	9.18 ± 0.38 ^a^
D2-UV/5.5	30.13 ± 0.15 ^ab^	14.53 ± 0.12 ^ab^	7.74 ± 0.02 ^b^
D3-UV/3.5	30.86 ± 0.61 ^a^	13.11 ± 1.45 ^ab^	9.19 ± 0.24 ^b^
D3-UV/5.5	30.11 ± 0.25 ^ab^	14.97 ± 0.01 ^ab^	7.87 ± 0.18 ^b^

* All values are expressed as means ± standard deviation (n = 3). Different letters per column indicate significance by means according to Tukey test (*p* < 0.05). UP, unprocessed; D1-UV, 16.5 mJ/cm^2^; D2-UV, 27.7 mJ/cm^2^; D3-UV 40 mJ/cm^2^.

**Table 3:** Total, simple sugars, and Aloin A content of *A. vera* –pitaya blend associated with different pH values and UV-C irradiation process.

Treatment/pH	Total Sugars (mg/g dm)	Glucose (mg/g dm)	Fructose (mg/g dm)	Sucrose (mg/g dm)	Aloin A (mg/L)
UP/3.5	839.11 ± 11.78 ^ab^	25.31 ± 0.04 ^c^	27.17 ± 0.10 ^c^	128.92 ± 0.32 ^e^	2.84 ± 0.85 ^a^
UP/5.5	807.52 ± 1.93 ^cd^	23.07 ± 0.05 ^e^	25.44 ± 0.02 ^d^	120.66 ± 0.58 ^f^	2.95 ± 0.23 ^a^
D1-UV/3.5	789.57 ± 5.17 ^d^	27.50 ± 0.03 ^b^	28.28 ± 0.21 ^b^	132.28 ± 0.19 ^cd^	3.99 ± 0.12 ^a^
D1-UV/5.5	846.91 ± 15.03 ^a^	24.63 ± 0.03 ^d^	26.06 ± 0.18 ^d^	131.08 ± 0.15 ^d^	3.05 ± 0.31 ^a^
D2-UV/3.5	796.43 ± 3.55 ^d^	27.68 ± 0.08 ^b^	28.84 ± 0.19 ^ab^	134.75 ± 0.30 ^b^	3.36 ± 0.12 ^a^
D2-UV/5.5	838.44 ± 1.47 ^ab^	27.48 ± 0.08 ^b^	28.93 ± 0.19 ^ab^	132.56 ± 0.23 ^c^	2.95 ± 0.71 ^a^
D3-UV/3.5	819.19 ± 2.34 ^abcd^	27.92 ± 0.03 ^a^	29.45 ± 0.15 ^a^	137.86 ± 0.28 ^a^	2.58 ± 0.46 ^a^
D3-UV/5.5	810.64 ± 8.16 ^bcd^	27.48 ± 0.03 ^b^	28.87 ± 0.27 ^ab^	133.28 ± 0.28 ^c^	2.67 ± 0.44 ^a^

Means ± standard deviation (n = 4). Different letters per column are significantly different according to Tukey test (*p* < 0.05). UP, unprocessed; D1-UV, 16.5 mJ/cm^2^; D2-UV, 27.7 mJ/cm^2^; D3-UV 40 mJ/cm^2^.
